# The ketogenic diet in Parkinson’s disease: mechanisms, evidence, and clinical practice

**DOI:** 10.3389/fnut.2026.1918711

**Published:** 2026-09-17

**Authors:** Mula A, Yan Bai, Yulin Wang, Peizhu Lv, Shun Wang

**Affiliations:** 1 The Second Affiliated Hospital, Heilongjiang University of Chinese Medicine, Harbin, China; 2 Institute of Acupuncture and Moxibustion, Heilongjiang Academy of Traditional Chinese Medicine, Harbin, China

**Keywords:** gut-brain axis, ketogenic diet, metabolic intervention, neuroprotection, Parkinson’s disease, α-synuclein

## Abstract

Parkinson’s disease (PD) represents the second most common neurodegenerative disorder across the globe. Conventional levodopa treatment can only alleviate disease-related symptoms and fails to restrain the progressive deterioration of PD. Metabolic abnormalities that mainly include mitochondrial dysfunction, cerebral insulin resistance, and disrupted energy homeostasis have been proven to be core pathogenic factors of PD, which makes metabolic interventions a promising therapeutic strategy for disease management. The ketogenic diet (KD) is a high-fat, low-carbohydrate dietary regimen that can induce endogenous ketone synthesis, and it exerts multi-dimensional neuroprotective effects on PD. This review systematically elaborates the potential molecular mechanisms through which KD protects against PD, which covers the improvement of cerebral energy metabolism, antioxidant and anti-apoptotic activities, suppression of neuroinflammation, regulation of α-synuclein (α-syn) pathological aggregation, and modulation of gut-brain axis. We comprehensively synthesize preclinical research evidence from cell and animal models as well as clinical data across case reports, pilot studies and randomized controlled trials, and we summarize critical clinical application principles that include dietary protocols selection, patient screening criteria, safety monitoring indicators and diet-drug interactions. Although KD intervention appears to improve selected motor and non-motor symptoms of PD, the current evidence remains preliminary, with prominent limitations including inconsistent trial outcomes, high dropout rates, heterogeneous dietary schemes and insufficient exploration of multi-omics. The inconsistent clinical outcomes, high trial dropout rates, heterogeneous dietary protocols, limited biomarker validation, and insufficient long-term efficacy and safety data limit its widespread clinical application. Large-scale, well-controlled clinical trials with standardized ketosis monitoring, disease-progression biomarkers, and extended follow-up are therefore required to verify the translational application value of KD in PD management.

## 1 Introduction

Parkinson’s disease (PD) is the second most prevalent neurodegenerative disease following Alzheimer’s disease (1). It affects more than 10 million individuals worldwide, with prevalence increasing steeply with age, reaching 9.34 cases per 1000 individuals aged 60 years or older (2, 3). The clinical manifestations of PD consist of core motor symptoms that include bradykinesia, resting tremor, muscular rigidity and postural instability, as well as a variety of non-motor symptoms that involve constipation, cognitive impairment, sleep disorders and depression, which usually appear several years earlier than motor symptoms and severely affect the quality of life of PD patients (4, 5).

Levodopa-mediated dopamine replacement therapy serves as the mainstream pharmacological treatment for PD at present. This treatment can effectively relieve clinical symptom but cannot repair damaged neurons, and long-term medication is often accompanied by adverse reactions such as motor fluctuations, dyskinesias, and the “wearing-off” phenomenon (6, 7). Accumulating research evidence confirms that metabolic disorders run through the entire pathological process of PD. Mitochondrial complex I deficiency in the substantia nigra leads to insufficient ATP synthesis and excessive accumulation of reactive oxygen species (ROS) (8, 9), cerebral insulin resistance impairs neuronal survival and synaptic plasticity (10, 11), and gut microbiota dysbiosis promotes neuroinflammation and α-synuclein (α-syn) propagation via the gut-brain axis (12). These research findings provide a solid theoretical basis for the clinical application of metabolic intervention strategies in PD.

The ketogenic diet (KD) is a high-fat and ultra-low-carbohydrate dietary regimen that was first proposed for the treatment of refractory epilepsy in the 1920s (13, 14). The core mechanism of KD is to restrict carbohydrate intake so as to shift the body’s main energy metabolism pathway from glucose utilization to fatty acid oxidation, which further induces the production of ketone bodies including β-hydroxybutyrate (BHB), acetoacetate and acetone as alternative energy substances (4). In recent years, the application value of KD in neurodegenerative diseases treatment has attracted extensive research attention (15–17). Existing studies have verified that KD can not only optimize cerebral energy metabolism but also exert neuroprotective effects via antioxidant, anti-inflammatory, autophagy-regulating and gut microbiota-modulating mechanisms (18).

This review systematically summarizes the therapeutic potential of KD in PD, elaborates its underlying molecular mechanisms, integrates preclinical and clinical research evidence, and discusses key practical issues in clinical promotion, so as to provide references for subsequent translational research and clinical application of KD in PD treatment.

## 2 Molecular mechanisms of KD-mediated neuroprotection

### 2.1 Improvement of cerebral energy metabolism and mitochondrial function

Mitochondrial dysfunction is a core pathological feature of PD that is prominently observed in dopaminergic neurons of the substantia nigra. KD can effectively improve mitochondrial function by bypassing impaired complex I via a complex II-dependent mechanism, enhancing complex IV activity, and increasing ATP synthesis efficiency (19, 20). Ketone bodies represented by BHB provide a compensatory energy pathway: they bypass impaired complex I, are converted into acetyl-CoA entering the TCA cycle (21, 22), and generate fewer ROS per unit of energy produced compared to glucose, achieving dual protective effects on energy metabolism and redox homeostasis (17, 23).

In addition, BHB can activate the peroxisome proliferator-activated receptor γ coactivator 1α (PGC-1α) signaling pathway, which promotes mitochondrial biogenesis and optimizes mitochondrial morphology and physiological function (24). Preclinical evidence has further suggested that ketone bodies may facilitate mitochondrial quality control by through PINK1/Parkin-mediated mitophagy, a process that selectively eliminates dysfunctional mitochondria and inhibits the opening of the mitochondrial permeability transition pore (mPTP), thus preserving mitochondrial membrane potential and prevents the release of pro-apoptotic factors (25, 26). Nevertheless, a direct causal link between the ketogenic diet and activation of this specific pathway *in vivo* has not been firmly established and warrants further investigation. In MPTP-induced PD mouse models, medium-chain triglyceride ketogenic diet (MCT-KD) can significantly reduce mitochondrial loss, improve ATP synthesis and protect dopaminergic neurons from damage (27).

### 2.2 Antioxidative and anti-apoptotic effects

Oxidative stress is the pivotal pathological mechanism that induces dopaminergic neuronal death in PD (28). Under ketogenic metabolic conditions, BHB exerts indirect antioxidant effects by suppressing mitochondrial ROS production and activating the nuclear factor erythroid 2-related factor 2 (Nrf2) signaling pathway, which further upregulates the expression of antioxidant enzymes such as superoxide dismutase 2 (SOD2) and catalase (15, 20, 29, 30). Furthermore, through indirect modulation of cellular redox homeostasis, BHB metabolism can elevate the cytoplasmic NADPH/NADP^+^ ratio, thereby providing reducing equivalents for the glutathione and thioredoxin antioxidant systems and enhancing the overall antioxidant capacity of neurons (26). In MPTP-induced PD mouse models, MCT-KD exerts antioxidant effects via the PI3K/Akt/Nrf2 signaling pathway, reversing oxidative stress damage in dopaminergic neurons (27).

Additionally, findings from preclinical models indicate that KD may modulate the expression of apoptosis-related proteins. Specifically, studies have reported that KD upregulates the anti-apoptotic protein Bcl-2, downregulates the pro-apoptotic protein Bax, and inhibits the activation of caspase-1, caspase-3, and caspase-9, thereby blocking the neuronal apoptotic cascade (15, 27). In parallel, BHB has been shown to suppress caspase-1 activity, a key effector of pyroptosis, in experimental settings, which may further reinforce the anti-apoptotic and anti-inflammatory effects associated with KD (20).

### 2.3 Suppression of neuroinflammation

Chronic neuroinflammation plays a pivotal part in the initiation and progression of PD. Excessively activated microglia can release pro-inflammatory cytokines including TNF-α, IL-1β and IL-6, which directly damage dopaminergic neurons and accelerate α-syn aggregation (19, 31, 32). KD suppresses neuroinflammation through multiple synergistic regulatory pathways. Firstly, BHB has been well documented to inhibit NLRP3 inflammasome activation, primarily through post-transcriptional mechanisms, thereby reducing neuronal pyroptosis and the subsequent release of inflammatory factors IL-1β and IL-18. Studies in cellular models have reported the STAT3/NLRP3/GSDMD signaling axis as one cell-level downstream cascade contributing to microglial pyroptosis. However, this axis is not considered the primary mechanism through which BHB represses NLRP3 inflammasome activity (33–35). Secondly, BHB promotes the polarization of microglial from the pro-inflammatory M1 phenotype to the anti-inflammatory M2 phenotype, and this phenotypic transformation is closely associated with the metabolic reprogramming of microglia from glycolysis to oxidative phosphorylation (36). Thirdly, KD reduces intestinal permeability, which prevents gut-derived lipopolysaccharide from entering the systemic circulation and alleviates peripheral inflammatory responses (37, 38).

β-hydroxybutyrate can also inhibit the NF-κB signaling pathway to reduce the transcriptional expression of pro-inflammatory mediators (19). In addition to regulating microglial activity, BHB acts on astrocytes to upregulate EAAT1 expression via the Ca^2+^/CaMKII pathway, which enhances astrocytic glutamate uptake capacity and relieves neuroinflammation caused by excitotoxicity (39). The anti-inflammatory properties of KD are also mediated by metabolic sensors such as AMPK and mTOR, which induce anti-inflammatory metabolic phenotypes in glial cells (36).

In LPS-induced rat PD models, the anti-inflammatory effects of KD rely on the histone acetylation modification of the mGluR5 promoter region, which modulates the downstream Akt/GSK-3β/CREB signaling cascade (38). It is worth noting that preventive KD (KDp) intervention which is implemented before pathological changes occurs exhibits significantly better therapeutic effects than therapeutic KD (KDt) intervention which is conducted after symptom onset (38). This finding is consistent with the regulatory function of β-hydroxybutyrylation (Kbhb). As a novel histone post-translational modification induced by BHB, Kbhb links ketone metabolism to the transcriptional regulation of genes related to inflammation, chromatin remodeling and circadian rhythms (40).

### 2.4 Modulation of α-synuclein pathology

The misfolding and aggregation of α-syn to form Lewy bodies is the core pathological hallmark of PD (41, 42). KD may alleviate α-syn-related pathological damage through at least three synergistic mechanisms. Firstly, BHB may modulate α-syn folding and aggregation, although the precise molecular mechanisms underlying this effect remain unclear and require further investigation (43). Secondly, KD-induced ketosis activates SIRT1 and HIF-1α while inhibiting the mTORC1 complex, which enhances cerebral macroautophagy and facilitates the clearance of misfolded and aggregated α-syn proteins (13, 44). Thirdly, KD reduces α-syn aggregation in the enteric nervous system and optimizes gut-brain axis signal transmission. Although direct evidence for vagal transmission impairment is still insufficient, existing preclinical studies support the conclusion that KD can block the prion-like spread of α-syn from the gut to the central nervous system via the vagus nerve (37). Additionally, BHB maintains cellular protein homeostasis by modulating the Unfolded Protein Response (UPR), which alleviates endoplasmic reticulum stress and prevents the accumulation of damaged and aggregated proteins (40).

In aged rats, KD intervention can regulate the rhythmic expression of rSnca that encodes α-syn and rPark2 that encodes Parkin in the suprachiasmatic nucleus and substantia nigra, which indicates that KD can maintain circadian rhythm stability and delay the progression of α-syn-mediated neurodegeneration (45).

### 2.5 Regulation of the gut-brain axis: gut microbiota and metabolites as mediators

Gut-brain axis dysfunction is a key pathological event in the progression of PD (46, 47). PD patients typically exhibit obvious gut microbiota dysbiosis which is characterized by the depletion of short-chain fatty acid (SCFA)-producing bacteria, such as *Faecalibacterium* and *Roseburia*, and the enrichment of pro-inflammatory bacterial genera including *Escherichia* and *Shigella* (48, 49). Clinical microbial profiling studies have confirmed that the increased abundance of Enterobacteriaceae serves as a typical microbial signature of PD patients at baseline (50).

Ketogenic diet can effectively reshape the composition of gut microbiota in PD models and patients. In MPTP-induced PD mice, KD reverses gut dysbiosis by reducing the abundance of *Citrobacter*, *Desulfovibrio* and *Ruminococcus*, while increasing the abundance of *Dubosiella* (37). MCT-KD can further enrich beneficial bacterial genera including *Blautia* and *Romboutsia* in PD model mice (27). A 12-week clinical pilot study that enrolls 27 PD patients finds that the improvement of motor and non-motor symptoms after KD intervention is accompanied by specific microbial changes, which include the upregulation of *Enterococcus* and *Synergistota*, and the downregulation of *Alloprevotella* (50). These findings demonstrate that the therapeutic effects of KD in PD are closely related to the targeted remodeling of gut microbiota, which manifests as the enrichment of beneficial bacteria and the reduction of pro-inflammatory bacteria.

Ketogenic diet can also regulate the synthesis and secretion of microbiota-derived metabolites. It promotes the production of SCFAs represented by butyrate, which exerts anti-inflammatory and neuroprotective effects by activating GPR41/43 receptors and inhibiting histone deacetylase (51–53), and it reduces intestinal permeability to relieve systemic inflammatory responses (37). Notably, fecal microbiota transplantation from KD-treated mice can alleviate motor dysfunction and dopaminergic neuron loss in antibiotic-pretreated PD mice, which provides direct causal evidence that KD-modulated gut microbiota changes mediate neuroprotective effects (37) (Figure 1).

**FIGURE 1:**
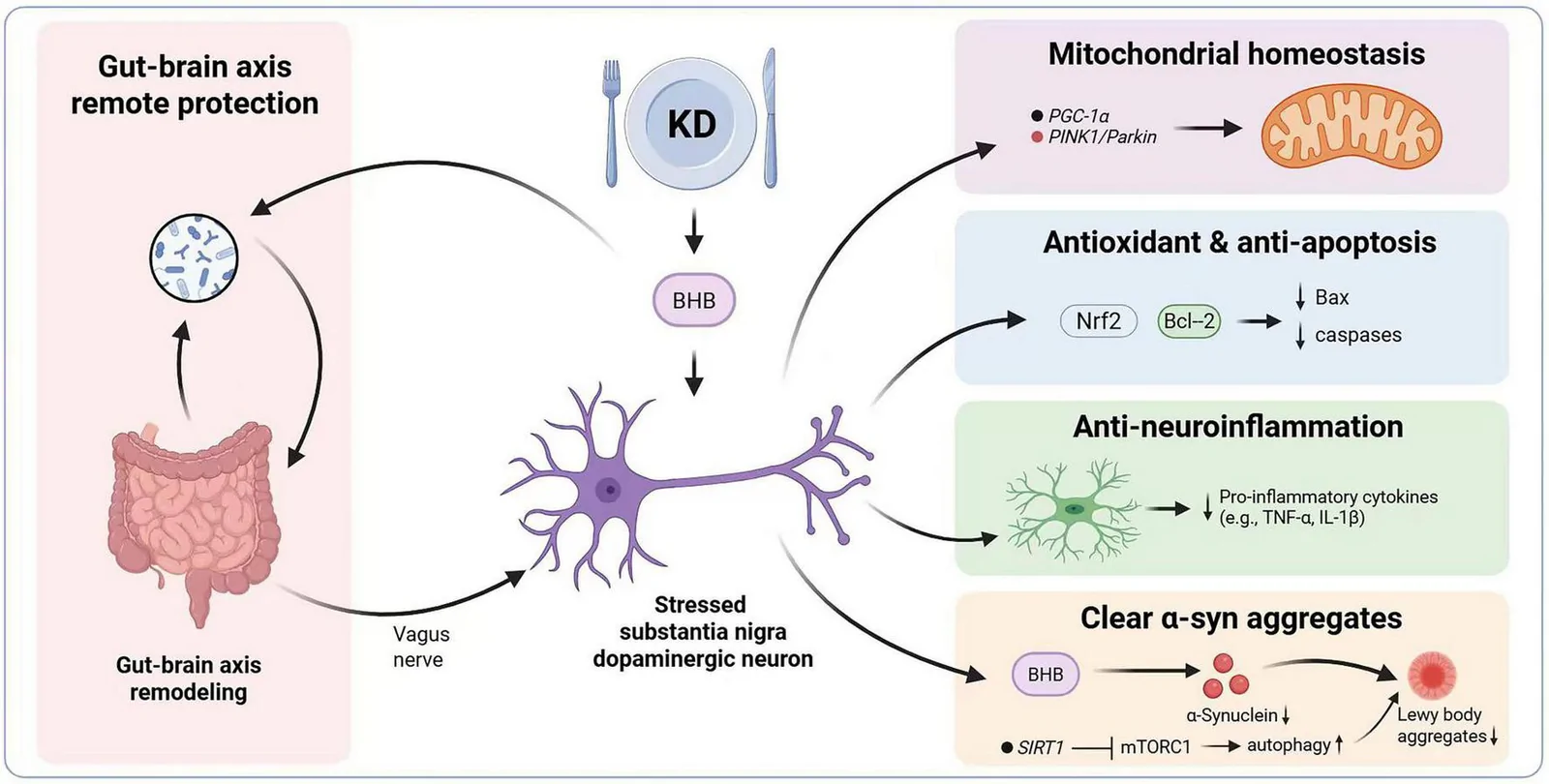
Molecular mechanisms of KD action in PD. Created with BioRender.com.

## 3 Preclinical evidence for KD in PD

### 3.1 Cell model studies

*In vitro* experimental studies have consistently verified the protective effects of ketone bodies against dopaminergic neuronal damage related to PD. Cellular models that are established with MPP^+^ stimulation in SH-SY5Y cells and primary neuronal cultures demonstrate that BHB treatment can effectively improve neuronal viability, reduce intracellular ROS accumulation, restore normal mitochondrial membrane potential, and inhibit the abnormal aggregation of α-syn (33). In BV2 microglial cells, BHB suppresses the activation of the STAT3/NLRP3/GSDMD signaling axis, which further inhibits microglial pyroptosis and reduces the release of pro-inflammatory cytokines including IL-1β and IL-18 (33).

Ferroptosis is an iron-dependent programmed cell death mode that is driven by intracellular lipid peroxidation and serves as a critical pathological mechanism of dopaminergic neuronal injury in PD. In MPP^+^-stimulated SN4741 dopaminergic neurons, BHB can upregulate the expression of zinc finger protein 36 (ZFP36), which directly binds to and destabilizes acyl-CoA synthetase long-chain family member 4 (ACSL4) mRNA. The ZFP36/ACSL4 signaling axis identified in these studies represents a novel molecular mechanism through which BHB exerts anti-ferroptosis and neuroprotective effects in PD cell models (54).

### 3.2 Animal model studies

A large number of *in vivo* animal studies have explored the therapeutic efficacy of KD in PD, and the collective research outcomes are largely supportive with a small number of inconsistent findings. In MPTP-induced mouse model of PD, continuous KD intervention for 8 weeks can significantly ameliorate motor dysfunction, increase the number of tyrosine hydroxylase-positive dopaminergic neurons in the substantia nigra pars compacta, and mitigate inflammatory responses in both brain and colon tissues (37). MCT-KD exhibits superior neuroprotective effects with multi-target regulatory mechanisms, as it activates the PI3K/Akt/Nrf2 antioxidant signaling pathway to alleviate oxidative damage, reduces mitochondrial loss to maintain normal energy metabolism, and suppresses excessive microglial activation in brain tissues. Transcriptomic and metabolomic analyses further confirm that MCT-KD modulates purine metabolism in the substantia nigra and remodels the composition of gut microbiota by enriching beneficial bacterial genera such as *Blautia* and *Romboutsia* in PD model mice (27). These preclinical findings collectively demonstrate the multi-dimensional neuroprotective properties of KD in PD animal models.

Studies based on LPS-induced rat PD models confirm that both preventive and therapeutic KD interventions can relieve dopamine-deficient behavioral abnormalities and inhibit neuroinflammation, while preventive KD treatment that is implemented before the onset of pathological changes produces significantly better therapeutic effects than post-symptomatic therapeutic intervention (38). In aged rats with circadian rhythm disorders, KD can regulate the rhythmic expression of clock genes, immune-related genes, and PD pathogenic genes in the suprachiasmatic nucleus and substantia nigra, which verifies the regulatory effect of KD on circadian homeostasis to delay neurodegenerative progression (45).

Nevertheless, several preclinical studies have yielded negative results that suggest the limitations of KD treatment. In a 6-OHDA-induced early PD rat model, sustained hyperketonemia which was maintained for 3 weeks before model establishment and 4 weeks after modeling failed to protect dopaminergic neurons from degeneration. Although a slight improvement in locomotor activity and normalized dopamine turnover were observed, no significant neuroprotective effect was detected, which indicates that simple elevation of BHB concentration is not sufficient to achieve therapeutic effects and that comprehensive metabolic remodeling is required for KD-mediated neuroprotection (55).

## 4 Clinical evidence for KD in PD

### 4.1 Case reports

A 24 weeks clinical case study focuses on (56) a 68-year-old female patient with stage I PD defined by the Hoehn and Yahr scale, who receives traditional KD intervention with a macronutrient ratio of 70% fat, 25% protein and 5% carbohydrates. The results show that this dietary intervention significantly improves multiple metabolic biomarkers of the patient. The levels of HbA1c decrease from 6.7% to 5.6%, fasting insulin decreases from 18.6 to 5.6 mIU/L, triglycerides decrease from 127 to 58 mg/dL, C-reactive protein decreases from 1.29 to 0.64 mg/L, and body weight decreases from 202 to 172.4 lbs. In terms of psychological symptoms, the patient’s Parkinson’s Anxiety Scale scores decrease from 23 to 17 and CESD-R-20 depression scores decrease from 42 to 34. However, the total Unified Parkinson’s Disease Rating Scale (UPDRS) scores increase from 24 to 33 after 24 weeks of intervention, which indicates that KD fails to block the progression of motor symptoms in this patient (56).

### 4.2 Pilot studies

Multiple pilot clinical studies have verified the feasibility and therapeutic efficacy of KD intervention in PD patients. A 12-week pilot study that enrolls 16 adults PD patients adopts a low-carbohydrate and healthy-fat KD regimen, and the results confirm significant improvements in anxiety levels and UPDRS Part I scores that evaluate mental status, behavioral changes and mood, along with favorable changes in a variety of metabolic biomarkers (57). A 24-week longitudinal pilot study that includes 7 PD patients further proves that KD intervention can improve cognitive function, mood status, motor and non-motor symptoms, reduce pain and anxiety, and enhance patient’s self-reported quality of life. However, it should be noted that the observed improvement in motor function was evaluated solely through UPDRS Part II, which assesses patient-reported experiences of daily living and is inherently subjective; no objective motor assessment via UPDRS Part III was performed in this study, limiting the interpretation of its motor-related findings. (58). A 12-week mixed-methods feasibility study that recruits 12 PD patients implements a modified low-dairy KD intervention and achieves a 100% trial completion rate, while 75% of participants complete more than three-quarters of daily food diary records. The stable macronutrient intake and elevated serum BHB levels of participants confirm sustained nutritional ketosis throughout the intervention period. Clinical analysis shows improvements in total UPDRS scores, mini-Balance Evaluation Systems Test results, freezing of gait symptoms, and PD Questionnaire-39 scores reflecting quality of life. Notably, although UPDRS Part III motor function scores also showed improvement, this change did not reach statistical significance; nevertheless, the observed trend in motor function remains clinically noteworthy and warrants further investigation in larger cohorts. All participants achieve effective weight loss, and most patients do not experience aggravated constipation symptoms (59).

Another 12-week pilot study that enrolls 27 PD patients demonstrates that KD intervention can significantly improve motor symptoms assessed by MDS-UPDRS Part III and non-motor symptoms assessed by the Non-Motor Symptom Scale. The specific clinical benefits include relieved anxiety and depression, improved cognitive function and alleviated constipation. Microbial detection analysis shows that the clinical symptom improvement of patients is accompanied by characteristic gut microbial alterations, which verifies that gut microbiota remodeling mediates the therapeutic effects of KD on PD (50).

### 4.3 Randomized controlled trials

An 8-week pilot randomized controlled trial compares the efficacy of KD and low-fat diet in 47 PD patients, and the results show that both dietary interventions can reduce MDS-UPDRS scores. The KD group exhibits a significantly better improvement in UPDRS Part I scores that evaluate non-motor daily living experiences, with a 41% reduction from baseline compared with an 11% reduction in the low-fat diet group. The largest between-group differences are observed in urinary problems, pain sensation, fatigue degree, daytime sleepiness and cognitive function. No significant differences are found between the two group in UPDRS Parts 2–4 that assess motor function and disease complication (60).

A randomized feasibility trial adopts a two-phase research design to compare MCT-KD and standard diet in PD patients, which includes a 1-week in-hospital double-blind intervention phase and a 2-week home-based open-label intervention phase. Among 16 enrolled participants, the mean acceptability score reaches 2.3 out of 3. The severity of non-motor symptoms is significantly reduced at week 3 of intervention, and the improvement is more prominent in the KD group. Motor function measures including the Timed Up and Go test results and UPDRS Parts 2–4 scores did not differ significantly between groups (61). A 3-month randomized controlled trial that enrolls 74 PD patients with voice disorders finds that all indicators of the Voice Handicap Index are improved significantly in the KD group compared with the regular diet group, which indicates that KD can effectively improve the voice quality of PD patients (62).

A phase 2 randomized crossover trial enrolls 52 PD patients to compare two 8-week Mediterranean-ketogenic interventions, Mediterranean-ketogenic diet (MeDi-KD) and MCT-supplemented Mediterranean diet (MeDi-MCT), and an 8-week washout period is set between the two intervention phases. The trial results confirm that no intervention-related serious adverse events or significant changes in plasma lipid profiles. Nutritional ketosis is successfully achieved in 50% of participants during the MeDi-KD intervention, while only 3% of participants achieve ketosis during the MeDi-MCT intervention. The MeDi-MCT can significantly reduce patients’ MDS-UPDRS Part II and Part IV scores. However, it should be noted that neither the MeDi-KD nor the MeDi-MCT intervention produced significant improvements in MDS-UPDRS Part III, which objectively assesses motor function. The 37% participant dropout rate of the trial reflects obvious practical feasibility challenges of long-term dietary intervention (63).

A recent 6-month randomized controlled trial evaluates the efficacy of a personalized low-glycemic plant-rich ketogenic diet combined with intermittent fasting in 40 PD patients. The intervention group shows a significant improvement in UPDRS-III motor function scores, apathy symptoms, daily living ability and gastrointestinal function, and patients with inflammatory phenotypes obtain more prominent therapeutic benefit (64). The core clinical research findings are summarized in Table 1.

**TABLE 1:** Clinical evidence for KD in PD.

Study type	Sample size	Intervention	Duration	Ketosis achieved	Dropout rate	Adherence level	Adverse effects	Primary endpoint	Effect size	Overall study quality	Key findings	Direction of evidence	References
Pilot study	*n* = 27 enrolled, 16 completed	KD	12 weeks	Urine ketones +++ to +++++ daily threshold	40.7%	Low–medium	No severe adverse events	MDS-UPDRS Part III (motor) and NMSS (non-motor)	Medium; motor *p* < 0.001, non-motor *p* < 0.0001	Low–moderate	• Identifies baseline dysbiosis with elevated *Enterobacteriaceae* in PD patients • Significantly improves motor and non-motor symptoms • microbial shifts: increased *Enterococcus* and *Synergistota*, decreased *Alloprevotella*	Supportive (motor and non-motor symptoms; linked to gut microbiota)	(50)
Case study	*n* = 1 (68-year-old female, Hoehn and Yahr stage I)	Traditional KD with 70% fat, 25% protein, and 5% carbohydrate	24 weeks	Average blood ketone 0.5 mmol/L; sustained nutritional ketosis	0%	Very high (daily blood ketone + food app tracking)	Mild early fatigue; no severe AEs	Biomarkers, CESD-R depression, PAS anxiety, UPDRS I–III	Unavailable for single-case research;	Low	• Significantly improves metabolic biomarkers including HbA1c, CRP, triglycerides, and fasting insulin • Increases HDL cholesterol levels • Reduces anxiety symptoms with limited improvement in depression	Supportive (biomarkers and anxiety symptoms)	(56)
Pilot study	*n* = 16	LCHF/KD	12 weeks	14/16 maintained nutritional ketosis (>0.5 mmol/L); mean ketone 0.64 mmol/L	0%	Medium (14/16 reached ketosis target)	Mild GI upset, headaches, mood swings, constipation, thirst, leg cramps (transient, easily mitigated)	UPDRS, PAS anxiety, CESD-R-20 depression, metabolic biomarkers	Small; PAS *p* = 0.0079, UPDRS Part I *p* = 0.0129; depression NS	Low–moderate	• Significantly improves Parkinson’s Anxiety Scale scores and UPDRS Part I scores • Shows favorable trends in metabolic biomarkers	Supportive (non-motor symptoms)	(57)
Longitudinal pilot study	*n* = 7 (8 started, 1 dropped)	LCHF/KD	24 weeks	Sustained ketosis >0.5–2.0 mmol/L throughout	12.5%	Medium	Constipation (mitigated by water, magnesium, salt); no severe AEs	UPDRS I and II, CESD-R-20, PAS, biomarkers, QoL interviews	Small–medium; triglycerides *p* = 0.047, HbA1c *p* = 0.016, weight *p* = 0.024	Low	• Improves metabolic biomarkers, cognition, mood, and motor and non-motor symptoms • Reduces pain and anxiety • Enhances self-reported quality of life	Supportive (small sample)	(58)
Mixed methods feasibility study	*n* = 12	Modified low-dairy KD	12 weeks	All reached nutritional ketosis by week 4; urine BHB ≥ 3 mM/L	0%	High (75% recorded ≥75% food diaries; macro targets largely met)	Weight loss 1.6%–7.48%; mild constipation worsening in 4/12; non-significant TC/LDL increase	Feasibility and acceptability (primary); UPDRS, PDQ-39, FoG, mini-BEST (secondary)	Small–medium; 8/12 reached MCID in motor function, balance, FoG, QoL	Moderate	• Achieves 100% completion rate with 75% adherence to food diaries • Confirms sustained nutritional ketosis throughout the intervention • Improves total UPDRS scores, motor function, balance, freezing of gait, and quality of life • Induces weight loss in all participants	Supportive (feasibility and symptom improvement)	(59)
Pilot randomized controlled trial	*n* = 47 randomized, 44 initiated, 38 completed	Low-fat high-carbohydrate diet vs. KD	8 weeks	KD group: mean blood ketone 1.15 ± 0.59 mmol/L (physiological ketosis)	19.1%	Medium (daily meal check + blood ketone monitoring)	Low-fat group: excessive hunger; KD group: intermittent tremor/rigidity exacerbation (transient)	MDS-UPDRS Parts 1–4 (primary)	Small–medium; Part 1: KD -41% vs. low-fat -11%, *p* < 0.001	Moderate	• Both groups achieve MDS-UPDRS score reductions • KD group shows significantly greater improvement in non-motor experiences of daily living • Largest benefits for urinary problems, pain, fatigue, daytime sleepiness, and cognition • No between-group difference in motor symptom scores	Supportive (non-motor symptoms)	(60)
Randomized feasibility trial with a double-blind inpatient phase followed by an open-label at-home extension	*n* = 16 (15 completed)	MCT-KD vs. standard diet	3 weeks	KD group attained blood ketosis >0.5 mM by day 4; greater at week 3	6.3%	High (mean net carb <10% of energy)	Fatigue (5), headache/light-headedness (2), diarrhea (1); 1 serious AE (DVT/PE, judged unrelated to diet)	Feasibility and acceptability (primary); TUG mobility (secondary)	Small; TUG NS between groups; NMSS reduction greater in KD group	Moderate	• MCT-KD feasible and acceptable • non-motor symptom severity decreased at week 3, with greater improvement in KD group • Ketosis achieved by day 4; non-significant upward trend in plasma levodopa metabolites in the KD group • No between-group differences in motor function or cognitive tests	Partially supportive (non-motor symptoms)	(61)
Randomized controlled trial	*n* = 74 randomized, 68 completed	KD vs. regular diet	3 months	Not explicitly quantified (KD defined as ∼90% fat calories)	8.1%	Not reported	GI side effects (nausea, vomiting, diarrhea, poor feeding): 5 total (3 KD, 2 RD); 1 arrhythmia in RD	Voice Handicap Index (VHI-10)	Medium; KD VHI total: 21.2 → 5.3, *p* < 0.01; RD: 22.2 → 20.9, NS	Moderate	• Baseline voice handicap parameters did not differ between groups • All Voice Handicap Index parameters improve significantly in the KD group • KD may serve as an alternative approach to improve voice quality in PD patients	Supportive (voice quality)	(62)
Phase II randomized crossover trial with an 8-week washout period between phases	*n* = 52 randomized, 48 initiated, 41 completed at least 1 phase, 33 completed both phases	MeDi-KD vs. MeDi-MCT	16 weeks total (8 weeks per arm)	MeDi-KD: 50% fasting BHB > 0.5 mM; MeDi-MCT only 3% hit ketosis threshold	36.5%	Medium-high (MEDAS adherence measured)	Mild hypotension, transient tremor aggravation, indigestion	MDS-UPDRS Part II and IV	Medium; Part II *p* = 0.039, Part IV *p* = 0.044	Moderate-high	• Both interventions well tolerated with no significant lipid profile changes • High dropout rate of 37% and modest overall adherence • MeDi-MCT significantly reduces MDS-UPDRS Part II and Part IV scores • Only 50% of participants achieve nutritional ketosis during the Mediterranean-ketogenic diet arm	Partially supportive (MeDi-MCT for patient-reported outcomes)	(63)
Randomized controlled trial	*n* = 40	Personalized plant-rich low-glycemic ketogenic diet with intermittent fasting vs. standard care	6 months	Not formally quantified in paper	0%	Medium-high (24-h dietary recall + regular nutrition counseling)	Minor bloating, mild nutrient fluctuation	MDS-UPDRS III motor, apathy, GI function	Medium-large; *p* < 0.001	Moderate	• Intervention group shows significant UPDRS-III improvement compared with standard care • Delivers significant benefits for apathy, activities of daily living, and gastrointestinal function • Shows greater efficacy in patients with an inflammatory phenotype	Supportive (motor and non-motor symptoms)	(64)

## 5 Clinical practice considerations for KD in PD

### 5.1 Types and protocols of KD

A variety of KD protocols with different degrees of stringency can be applied to PD treatment. The classic KD is a strict high-fat and ultra-low-carbohydrate dietary regimen that has a fat-to-combined carbohydrate and protein ratio ranging from 3:1 to 4:1, and it is originally designed for the clinical treatment of refractory epilepsy (65). MCT-KD has become a research hotspot in PD intervention because it can induce stable nutritional ketosis without strict carbohydrate restriction. The caprylic acid (C8) contained in MCT has been shown to enhance mitochondrial function, an effect mediated primarily through the promotion of mitophagy and mitochondrial biogenesis rather than through direct antioxidant properties (27, 61, 66, 67). Importantly, this mechanistic action has also been demonstrated in striatal neurons, supporting its potential relevance to PD pathology (68). These mitochondrial benefits may contribute to improved clinical tolerability and long-term adherence to the dietary regimen. The flexible low-carbohydrate healthy-fat ketogenic diet (LCHF/KD) is also widely used in PD clinical research due to its superior longer-term sustainability for patients (57, 58). More recently, novel hybrid dietary regimens represented by the MeDi-KD have been developed, which integrate the anti-inflammatory and neuroprotective properties of the Mediterranean diet with the metabolic advantages of ketogenic intervention (63).

### 5.2 Patient selection and indications

While current clinical guidelines have not incorporated KD into the standard treatment system for PD, emerging research evidence suggests that specific patient populations can benefit from KD intervention. The suitable candidates for KD treatment include patients who have insufficient response to conventional pharmacotherapy or suffer from motor fluctuation complications (60), patients with severe non-motor symptoms such as anxiety, depression, cognitive decline and constipation (56, 60, 63), as well as PD patients combined with metabolic disorders such as insulin resistance and metabolic syndrome (56). Conversely, KD intervention is not applicable to patients with metabolic disorders that contraindicate high-fat intake, which include fatty acid oxidation disorders, inborn errors of fatty acid transport/β-oxidation and pyruvate metabolism and severe uncontrolled hyperlipidemia (69).

### 5.3 Safety monitoring and adverse effects

Ketogenic diet appears to exhibit acceptable short-term safety in PD patient populations based on the limited available data from small-scale pilot studies. However, it must be emphasized that PD-KD trials remain few in number, small in sample size, and heterogeneous in design, precluding definitive conclusions regarding long-term safety. No serious adverse events have been reported in these short-term pilot studies (63). Nevertheless, this observation should be interpreted with caution given the limited scope and duration of existing evidence. Common mild adverse reactions include gastrointestinal discomfort such as nausea and constipation, transient fatigue, weight loss, and occasional aggravated tremor or rigidity (60, 61). Notably, unintended weight loss has been identified as a major concern when the diet is not expertly supervised, particularly given that PD patients may already be at risk for malnutrition. It is necessary to conduct baseline and regular dynamic monitoring of renal function and electrolyte balance during intervention, and the monitoring indicators include serum BHB, electrolytes, uric acid and urinary calcium (59, 70). Additionally, PD patients have enlarged fasting gallbladder volume, and the high-fat characteristics of KD may further increase the risk of gallbladder-related complications (71). In this regard, international KD guidelines recommend follow-up assessments at 3, 6, and 12 months, and then annually, with the monitoring frequency adjusted according to individual patient needs and the presence of specific risk factors (72).

### 5.4 Adherence and feasibility challenges

Poor long-term adherence is the main obstacle to the clinical promotion and application of KD in PD treatment. Existing clinical trials show that the dropout rate of KD intervention can reach 37%, and only 33 out of 52 randomized participants complete all intervention stages in a recent crossover study (63). Qualitative research has clarified the key factors that affect patients’ adherence to KD intervention. The favorable factors that improve adherence include professional guidance from registered dietitians, companion supervision from caregivers and patients’ positive recognition of dietary regimens (59, 73). The adverse factors that hinder long-term adherence include motor dysfunction that affects patients’ self-care ability, disease-induced apathy, inconsistent family dietary habits, cognitive resistance to dietary changes and economic pressure (73). A multidisciplinary intervention team composed of neurologists, clinical dietitians, nurses and occupational therapists is required to solve adherence problems (73, 74). Targeted strategies including individualized meal planning, gradual dietary transition, behavioral intervention guidance and reasonable goal setting can effectively improve patients’ long-term compliance with KD intervention.

### 5.5 Diet-drug interaction considerations

Evidence on interactions between KD and antiparkinsonian medications remains limited and inconclusive, even for levodopa, the most widely studied agent to date. KD may modulate levodopa’s pharmacokinetic profile, yet relevant research yields conflicting findings. One feasibility trial detected non-significant fluctuations in plasma DOPAC and dopamine following KD intervention (61), while another study reported no meaningful shifts in levodopa pharmacokinetics (75). Few controlled analyses have explored how ketosis interacts with other common anti-Parkinson medicines such as COMT inhibitors, MAO-B inhibitors, dopamine agonists and amantadine. It thus remains unclear whether ketosis interferes with drug absorption, circulation, clinical efficacy or adverse event risk. Comprehensive pharmacological trials are therefore warranted to untangle these diet-drug relationships. In the interim, clinicians are advised to closely monitor patients for motor fluctuations, dyskinesia, orthostatic hypotension, hallucinations, excessive sleepiness, and gastrointestinal intolerance when initiating or escalating a ketogenic regimen.

## 6 Challenges and future directions

Although existing evidence supports the therapeutic potential of KD in PD, several limitations hinder its broad clinical translation. A growing body of preclinical investigations has identified multiple potential neuroprotective pathways through which KD may counteract PD-related pathological alterations, yet human trials have only observed transient motor and non-motor improvements, predominantly measured by subjective MDS-UPDRS scores, and have failed to provide objective evidence of disease-modifying activity. This critical gap, coupled with heterogeneous study designs, limited long-term data, and the absence of validated biomarkers, calls for a critical evaluation of the current evidence and a well-defined framework for future investigations. A consolidated summary of the key challenges and corresponding research priorities is provided in Table 2.

**TABLE 2:** Summary of challenges and future directions for KD research in PD.

Theme	Current challenges	Future directions
Clinical evidence and trial design	Available trials feature small sample sizes, short intervention durations, and substantial methodological heterogeneity. Objective neuroimaging and CSF biomarkers are lacking to differentiate symptomatic improvement from genuine disease-modifying effects. High participant dropout rates further hinder reliable result interpretation.	Conduct large-scale, multicenter randomized controlled trials adopting unified study protocols. Incorporate multimodal biomarkers including DAT-SPECT/PET, CSF α-syn, NfL, and quantitative MRI. Implement follow-up periods longer than 12 months to evaluate long-lasting therapeutic benefits and chronic safety profiles.
KD protocol standardization	Consensus has not been reached regarding the optimal KD regimen. Variations across classic KD, MCT-KD, LCHF, and MeDi-KD, alongside inconsistent fat-to-carbohydrate ratios, MCT contents and meal compositions, impair cross-study comparability.	Perform systematic head-to-head comparisons among major KD modalities. Evaluate multiple endpoints in parallel, including ketosis attainment, treatment adherence, therapeutic efficacy, and adverse event profiles.
Patient stratification and biomarkers	No validated predictors of KD response in PD; APOE ε4 evidence extrapolated from Alzheimer’s disease, not established in PD; impact of metabolic phenotype, insulin resistance, and gut microbiome composition unclear. The influences of metabolic phenotypes, insulin resistance status and gut microbiome composition on treatment outcomes remain poorly defined.	Deploy multi-omics profiling covering genomics, metabolomics and metagenomics to identify PD subgroups susceptible to dietary benefits. Carry out prospective validation for candidate predictive biomarkers.
Gut-brain axis mechanisms	Human studies cannot confirm the causal contributions of particular bacterial strains and microbial metabolites. It also remains uncertain whether gut microbial alterations persist after dietary discontinuation, and whether such microbial alterations correlate with clinical therapeutic responses.	Perform long-term microbiome monitoring. Deploy FMT and gnotobiotic experimental models to establish causal relationships. Analyze longitudinal correlations between gut microbial remodeling and clinical outcomes.
Mechanistic understanding	Overemphasis on BHB, with acetoacetate and acetone understudied; most mechanistic data from cell/animal models, lacking human multi-omics validation; unclear whether benefits stem from ketosis, dietary matrix, or synergistic effects.	Implement comparative trials between exogenous ketone supplementation and complete KD regimens. Apply human-based longitudinal multi-omics approaches incorporating transcriptomics, proteomics and metabolomics to verify relevant molecular pathways among PD patients.
Safety and long-term risks	Short-term safety is generally acceptable according to pilot-study observations. Nevertheless, chronic adverse outcomes encompassing cardiovascular complications, sarcopenia, osteoporosis, nephrolithiasis and hepatic steatosis have not undergone systematic monitoring. Unintentional body weight loss represents a prominent clinical risk in the absence of expert supervision.	Establish long-term safety registries. Carry out structured monitoring following international KD guidelines at 3-month, 6-month, 12-month intervals with subsequent annual reassessments. Deliver comprehensive nutritional support protocols for malnutrition prevention.
Diet-drug interactions	KD intervention may modify levodopa pharmacokinetic profiles, yet relevant research yields inconsistent conclusions. Research data regarding interactions with COMT inhibitors, MAO-B inhibitors, dopamine agonists and amantadine are absent.	Conduct dedicated pharmacokinetic and pharmacodynamic trials for all mainstream anti-PD agents. Formulate standardized co-administration guidelines.
Adherence and feasibility	Long-term adherence to KD intervention remains suboptimal. High dropout rates can be observed, and adherence barriers consist of motor dysfunction, apathy, family dietary habits, and financial constraints.	Apply digital monitoring tools and behavioral coaching. Develop culturally adapted meal plans and deliver integrated services from multidisciplinary support teams.
Evidence synthesis and negative findings	Overoptimistic interpretation may disregard ineffective or unfavorable research observations. The overall methodological quality of the existing evidence body is limited.	Apply systematic evidence-grading tools for evidence evaluation. Guarantee transparent reporting of neutral and null research findings. Maintain objective and balanced appraisal for the therapeutic potential of KD interventions.

### 6.1 Clinical evidence, trial design, and protocol standardization

Current clinical evidence suffers from notable heterogeneity and insufficient long-term observation (76). Most human pilot trials only last 8–12 weeks to 6 months and rely on subjective symptom ratings rather than multi-modal pathological biomarkers, making it impossible to differentiate temporary symptomatic relief from genuine disease-modifying effects (76). While KD consistently improves non-motor symptoms, its motor efficacy varies widely across studies, and high dropout rates disturb results interpretation (63). Short intervention cycles only capture mild acute adverse reactions, lacking systematic monitoring for chronic risks such as cardiovascular complications, sarcopenia, osteoporosis, micronutrient insufficiency, nephrolithiasis and hepatic steatosis (76). Furthermore, no consensus exists regarding the optimal KD protocol; variations in fat-to-carbohydrate ratios, macronutrient composition, and MCT content across studies reduce cross-study comparability. To address these deficiencies, future trials must adopt large-scale, multicenter randomized designs with unified protocols, incorporate multimodal objective biomarkers, extend follow-up beyond 12 months, and conduct systematic head-to-head comparisons across major KD modalities to identify the most effective and patient-tolerable regimen for different PD subgroups.

### 6.2 Patient stratification and predictive biomarkers

Few studies clarify how intrinsic patient traits, including genetic backgrounds, metabolic phenotypes, insulin resistance status, and gut microbiome composition modulate KD therapeutic responses. Notably, evidence regarding the APOE ε4 genotype as a predictive biomarker derives primarily from Alzheimer’s disease research; specific data in PD are currently lacking, and this remains an extrapolation rather than an established fact (76). Without validated multi-omics signatures for patient stratification, uniform KD regimens cannot enable personalized intervention. Future research should prioritize multi-omics profiling to identify predictive signatures of KD response, with clear distinction between established PD-specific evidence and extrapolations from other disease contexts, and validate candidate biomarkers prospectively in well-characterized patient cohorts.

### 6.3 Mechanistic gaps: from BHB to the full dietary matrix

Existing mechanistic research predominantly focuses on BHB, while acetoacetate and acetone remain understudied despite metabolomic data confirming their altered levels in PD patients (77, 78). It remains unclear whether KD’s clinical benefits stem from ketosis alone, synergistic effects of the full dietary formula, or independent contributions of high-fat substrates and altered micronutrient intake. Importantly, isolated ketone supplement trials cannot replicate the multi-target neuroprotective effects observed with complete KD intervention, indicating that diet composition exerts therapeutic effects beyond ketone production alone. Nearly all mechanistic conclusions derive from cell and animal experiments; human tissue validation and multi-omics verification remain scarce. To address these gaps, future investigations should prioritize human-based longitudinal multi-omics trials integrating inflammatory, mitochondrial, and oxidative stress markers with metabolomic, proteomic, and transcriptomic profiling, thereby validating KD’s regulatory pathways directly in PD patients. Comparative trials between exogenous ketone supplementation and complete KD regimens are also warranted to disentangle the independent contributions of individual ketone bodies versus the full dietary matrix.

### 6.4 Safety, adherence, and practical challenges

Ketogenic diet exhibits acceptable short-term safety in PD populations based on limited pilot data, but unintended weight loss is a major concern without expert supervision. The high-fat nature of KD may increase gallbladder-related risks in PD patients, who already have enlarged fasting gallbladder volume (71). poor long-term adherence remains a critical obstacle, driven by motor dysfunction, disease-related apathy, family dietary habits, and financial constraints (73). Diet-drug interactions remain poorly characterized beyond levodopa; no data exist for COMT inhibitors, MAO-B inhibitors, dopamine agonists, and amantadine. Future efforts should standardize the implementation of international KD monitoring guidelines, with follow-up assessments at 3, 6, and 12 months and annually thereafter, along with nutritional support protocols to prevent unintended weight loss and malnutrition. Digital monitoring tools, behavioral coaching, and culturally adapted dietary schemes should be developed to improve long-term patient compliance. Dedicated pharmacokinetic interaction trials are also needed to systematically evaluate how ketosis affects the absorption, metabolism, clinical efficacy, and adverse event risks of all mainstream anti-Parkinson medications, rather than focusing exclusively on levodopa.

While many reports emphasize the “promising therapeutic potential” of KD, a balanced appraisal requires acknowledgment of negative and null findings. In the pilot randomized controlled trial, although KD improved non-motor symptoms, some participants experienced transient worsening of tremor and rigidity, and no significant between-group differences were observed for motor function (60). A systematic review concluded that the overall evidence quality is low, with most studies being small, unblinded, and lacking objective outcome measures (76). These findings counterbalance the optimistic narrative and underscore the need for rigorous, well-controlled trials before KD can be recommended as a routine adjunctive therapy. Future evaluations should adopt standardized evidence-grading systems to ensure transparent inclusion of negative findings and cautious interpretation of preliminary results, preventing overstatement of KD’s therapeutic potential in the absence of robust confirmatory data.

## 7 Discussion and conclusion

Despite its theoretical plausibility, the current evidence supporting KD as a therapeutic intervention for PD remains preliminary and insufficient for routine clinical use. Preclinical studies have demonstrated that ketone bodies, particularly BHB, engage multiple pathways relevant to PD pathophysiology, including mitochondrial bioenergetics, oxidative stress reduction, neuroinflammation suppression, α-syn proteostasis, and gut microbiota remodeling. When translating these mechanistic findings to clinical reality, however, a more nuanced picture emerges. The most consistent benefit observed across pilot trials is the improvement of non-motor symptoms, including cognitive function, anxiety, depression, constipation, and speech impairment. In contrast, motor outcomes remain uncertain and highly variable. While some studies reported UPDRS Part III improvements, others found no significant motor benefit, and at least one trial documented transient worsening of tremor and rigidity in a subset of participants. This inconsistency, coupled with the reliance on subjective rating scales rather than objective motor assessments in several studies, precludes definitive conclusions regarding KD’s motor effects.

Methodological limitations further constrain the interpretability of existing evidence and underscore the central unresolved question in the field. Most trials are small, with sample sizes below 50 participants, short-term with durations of 8–12 weeks, and open-label, with dropout rates up to 37%. Objective biomarkers of disease progression, such as DAT-SPECT/PET for dopaminergic integrity, CSF α-synuclein, neurofilament light chain, and quantitative MRI, have not been systematically incorporated. Consequently, whether KD exerts genuine disease-modifying effects or merely provides transient symptomatic relief remains unknown. From a clinical standpoint, practical considerations further temper enthusiasm. KD requires expert supervision to prevent unintended weight loss and nutritional deficiencies, may exacerbate gallbladder-related risks in PD patients, and faces substantial long-term adherence barriers. Drug-diet interactions remain inadequately characterized beyond levodopa. Several exploratory research directions, including time-restricted KD, anti-ferroptosis mechanisms, Kbhb epigenetic modifications, multi-omics patient stratification, and AI-driven objective monitoring, may advance precision ketogenic therapy but remain preliminary at present.

In conclusion, the clinical evidence for KD in PD, while mechanistically plausible and supported by preliminary data showing consistent non-motor benefits, remains insufficient to inform routine practice. The discrepancy between robust preclinical mechanisms and inconsistent clinical outcomes, particularly regarding motor function, highlights the urgent need for larger, biomarker-driven, long-term trials. Whether KD can slow PD progression or merely relieve transient symptoms remains the most critical unanswered question. At present, KD should be viewed as an experimental intervention rather than an established adjunctive therapy, and its use should be strictly limited to supervised research settings. Future research must prioritize predictive biomarker identification for patient stratification, adherence-enhancing strategies, and ultimately, rigorous randomized controlled trials to determine whether KD can be translated into a validated clinical tool for PD.
